# Effects of Short- and Long-Term Variation in Resource Conditions on Soil Fungal Communities and Plant Responses to Soil Biota

**DOI:** 10.3389/fpls.2018.01605

**Published:** 2018-11-06

**Authors:** Philip G. Hahn, Lorinda Bullington, Beau Larkin, Kelly LaFlamme, John L. Maron, Ylva Lekberg

**Affiliations:** ^1^Division of Biological Sciences, University of Montana, Missoula, MT, United States; ^2^MPG Ranch, Missoula, MT, United States; ^3^Department of Ecosystem and Conservation Sciences, University of Montana, Missoula, MT, United States

**Keywords:** arbuscular mycorrhizal fungi, context-dependent, drought stress, intraspecific variation, plant-soil feedback, plant defense, plant traits, soil fungi

## Abstract

Soil biota can strongly influence plant performance with effects ranging from negative to positive. However, shifts in resource availability can influence plant responses, with soil pathogens having stronger negative effects in high-resource environments and soil mutualists, such as arbuscular mycorrhizal fungi (AMF), having stronger positive effects in low-resource environments. Yet the relative importance of long-term vs. short-term variation in resources on soil biota and plant responses is not well-known. To assess this, we grew the perennial herb *Asclepias speciosa* in a greenhouse experiment that crossed a watering treatment (wet vs. dry treatment) with a manipulation of soil biota (live vs. sterilized soil) collected from two geographic regions (Washington and Minnesota) that vary greatly in annual precipitation. Because soil biota can influence many plant functional traits, we measured biomass as well as resource acquisition (e.g., root:shoot, specific leaf area) and defense (e.g., trichome and latex production) traits. Due to their important role as mutualists and pathogens, we also characterized soil fungal communities in the field and greenhouse and used curated databases to assess fungal composition and potential function. We found that the experimental watering treatment had a greater effect than soil biota origin on plant responses; most plant traits were negatively affected by live soils under wet conditions, whereas responses were neutral or positive in live dry soil. These consistent differences in plant responses occurred despite clear differences in soil fungal community composition between inoculate origin and watering treatments, which indicates high functional redundancy among soil fungi. All plants grown in live soil were highly colonized by AMF and root colonization was higher in wet than dry soil; root colonization by other fungi was low in all treatments. The most parsimonious explanation for negative plant responses in wet soil is that AMF became parasitic under conditions that alleviated resource limitation. Thus, plant responses appeared driven by shifts *within* rather than *betwee*n fungal guilds, which highlights the importance of coupling growth responses with characterizations of soil biota to fully understand underlying mechanisms. Collectively these results highlight how short-term changes in environmental conditions can mediate complex interactions between plants and soil biota.

## Introduction

Soil biota can strongly influence plant performance (Richardson et al., 2009; Berendsen et al., 2012; van der Putten et al., 2013). For example, ubiquitous arbuscular mycorrhizal fungi (AMF) colonize roots of approximately 75% of vascular plant species (Brundrett, 2009) and often benefit plants by facilitating their nutrient acquisition, protecting them from pathogen attack, or enhancing their drought tolerance (Smith and Read, 2008). However, colonization by AMF is not always a net positive for plant hosts, as these fungi can also act in a more parasitic fashion under certain circumstances (Johnson et al., 1997; Klironomos, 2003; Grman, 2012). Furthermore, some non-mycorrhizal fungi are well-known pathogens that can have strong negative impacts on their plant hosts (Raaijmakers et al., 2009). While these effects are well documented, it is also increasingly clear that the direction and magnitude of plant-soil biota interactions are extremely context dependent. A major challenge, therefore, is to understand what variables influence components of the soil biota community (e.g., AMF, fungal pathogens) and whether these changes affect plant responses to soil biota.

Resource availability in soils (e.g., nutrients or water availability) is thought to influence both attributes of the soil microbial community (Johnson, 1993; Leff et al., 2015) and how this community influences plants (Cook and Papendick, 1972; Johnson et al., 1997; Revillini et al., 2016). It is typically expected that mutualistic elements of soil biota are more beneficial and possibly more abundant in stressful, low-resource environments (Treseder, 2004; Johnson et al., 2010; Grman, 2012), because under these circumstances plants benefit by allocating more resources (carbon) to these symbionts which in-turn helps hosts acquire limiting resources or better tolerate various stressors. Soil pathogens, on the other hand, are thought to be more harmful in benign, high-resource environments, because they are more abundant under these conditions (Tompkins et al., 1992; Reynolds et al., 2003; Bell et al., 2006; Hersh et al., 2012; Veresoglou et al., 2012), and because plant hosts tend to be less defended against various antagonists in high-resource environments (Coley et al., 1985). Empirical support for these ideas comes from studies that have sampled soil biota from long-term fertilization plots and found them to be less beneficial to plants than soil biota collected from unfertilized soils (Johnson et al., 2010; Revillini et al., 2016).

Despite broad patterns in how nutrients may influence soil biota and plant responses, less is understood about how short-term changes in water availability, as occurs due to bouts of precipitation or drought, favor particular groups of soil biota and drive rapid shifts in their function. For example, AMF isolates from dry environments are better able to improve plant water relations than isolates from more mesic environments (Stahl and Smith, 1984), and a microbial community with previous exposure to drought is more beneficial to drought-stressed plants than a microbial community with no history of drought (Lau and Lennon, 2012). However, most studies that document effects of soil microbial communities and plant responses do so under either short-term or long-term conditions, but not both (e.g., Johnson et al., 2010; Lau and Lennon, 2012, but see Evans and Wallenstein, 2012; Zeglin et al., 2013; Kaisermann et al., 2017). Thus, an additional question concerns the relative importance of long-term differences in resource availability among locations, vs. short-term changes in resource conditions within sites on soil biota and their function.

**Figure 1:**
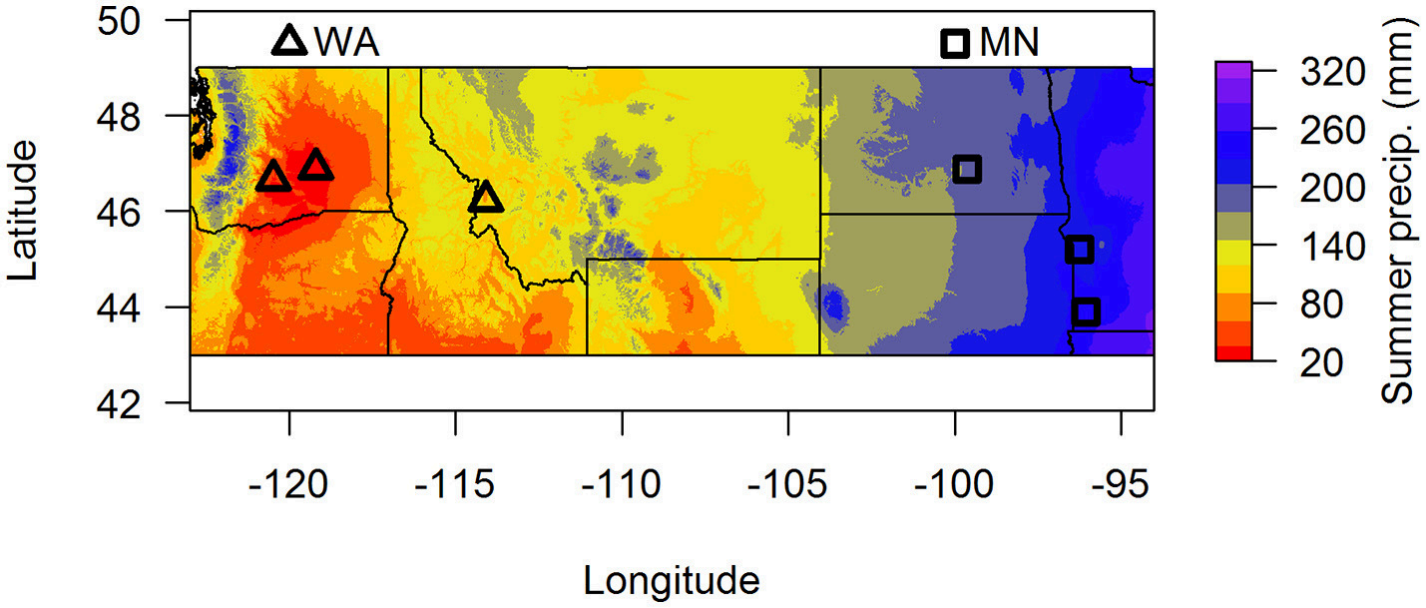
Map of soil collection locations. Summer precipitation (mm) is 30-year averages from the BioClim database. Samples were pooled by region (dry = WA, wet = MN) for the greenhouse experiment.

In this paper, we used two complementary approaches to address these questions. First, we characterized fungal communities in soil in the top 15 cm from around the perennial herb *Asclepias speciosa* from two geographic regions that vary greatly in summer precipitation (Washington and Minnesota, Figure 1). We then grew *A. speciosa* in either live or sterilized soil from each of the two regions under well watered or drought conditions and measured plant functional traits and plant responses to soil biota depending on soil origin and water availability. We also characterized changes in the soil fungal community based on geographic origin of soil biota and watering treatment. We focused on soil fungi because they are one of the most important groups of soil-borne pathogens (Raaijmakers et al., 2009) and mutualists (Smith and Read, 2008). We predicted that fungal communities would differ between the two regions and that the relative abundance of pathogens would be greater in sites with higher precipitation. We also predicted that plants grown in wet soils in the greenhouse would experience more negative responses to soil biota than plants grown in dry soils, and that those negative responses would be greater in soil originating from wetter areas.

## Materials and methods

### Plant material

*Asclepias speciosa* is a perennial herbaceous plant distributed throughout much of western North America. This plant is highly responsive to AMF (Busby et al., 2011); traits related to growth or resource acquisition (biomass, specific leaf area, root:shoot) and defense (latex and trichomes; Agrawal and Fishbein, 2006) all respond to AMF (Waller et al., 2018). Because *A. speciosa* traits vary among populations distributed across environments gradients (Waller et al., 2018), we collected seeds from five populations spanning the entire resource gradient over which we sampled soils to capture the range of traits values representative of this species (Table S1). Within each population, we haphazardly collected one seed pod (i.e., follicle) from 4 to 5 different ramets. All seeds were collected in September 2015. *Asclepias* are mostly self-incompatible and pollinated mainly by insects (mainly Hymenoptera). Pollen are transferred in packets (i.e., pollinium) such that all propagules within a fruit are full siblings.

### Soil collection for fungal community characterization, soil nutrients, and greenhouse experiment

To assess whether soil fungal communities differed between the two regions, we identified three sites at the western end (two sites in Washington and one site in Montana) and three sites at the eastern end of *A. speciosa's* distributional range (two sites in Minnesota, and one in North Dakota, Figure 1). Within each site, we collected soil (0–15 cm depth) from around 10 *A. speciosa* plants that were at least 5 m apart. Sites differ substantially in precipitation regimes (30-year averages), with sites in MN receiving about three times the amount of summer precipitation than sites in WA (Figure 1 and Table S1). Approximately 10 mL of soil from each soil sample was placed in a small envelope and immediately dried using desiccant. This soil sample was ultimately used for soil fungal DNA extraction. The remaining soil samples were pooled within sites, sieved through a 2 mm sieve and air dried. Some of this soil was used for analyses of macro and micronutrients (Ward Laboratory Inc., Kearney, NE, United States). The rest was kept cool and ultimately used as inoculum in the greenhouse experiment (which was started within 2 weeks of soil collections). Soil inocula used in the greenhouse experiment were pooled across the three sites within a region, resulting in two sources of inocula. These pooled samples were either sterilized via autoclaving (3 sessions × 90 min per session) or not. We refer to these two regions as WA and MN for brevity hereafter, since the majority (2 of 3) of regional samples originated from these states.

Sampling design and analyses associated with assessing soil biota effects in plant-soil feedback experiments has received considerable attention recently (e.g., Reinhart and Rinella, 2016; Cahill et al., 2017; Gundale et al., 2017). Although pooling of samples reduces degrees of freedom and limits generalizations, we pooled our soil inocula within regions because we were primarily interested in testing the effects of short-term watering treatments on changes in soil biota community composition and responses of plant traits to live soil biota. As such, we considered the individual pot receiving the various soil inocula as the relevant replicate, not the individual soil samples collected within sites in the field. We recognize that this pooling approach limits our ability to robustly determine how soil origin influences plant responses to soil biota. However, we note that for comparisons of fungal community composition across sites, we relied on molecular analyses conducted on individual soil samples collected from the soils (0–15 cm deep) around 10 individual plants within each site (see below).

### Greenhouse experiment

On 13–20 July, 2016, we germinated *Asclepias* seeds in water and planted them in 550 ml Deepots (Stuewe and Sons, Inc., Tangent, OR, United States) in the University of Montana's greenhouse. We used between 1 and 28 individual plants from 2 to 6 full-sibling families from each of five populations, resulting in a total of 208 plants that survived until the end of the experiment. Pots contained 100 mL of sand topped with 400 mL of a 1:1:1 mixture of sand, turface and sterilized field soil mix (see Table 1 for soil characteristics). To each pot, we added 50 mL of either WA or MN live or sterile inocula, placed as a layer approximately 5 cm from the soil surface for a total of 25 replicates of each watering treatment (wet or dry) × soil biota (live or sterile) × inoculum origin (MN or WA) combination.

**Table 1:** Soil nutrient analysis from soil inoculate and background soil used in the greenhouse experiment.

**Soil type**	**1:1 Soil pH**	**Organic matter LOI %**	**CEC/Sum of cations me/100 g**	**Nitrate-N ppm N**	**Potassium ppm K**	**Mehlich P-III ppm P**
WA (Dry origin), Sterile pooled inoculate (10% Volume)	7.8	3.3	21.7	7.0	638	46
WA (Dry origin), Live pooled inoculate (10% Volume)	7.9	3.8	22.0	9.5	592	31
MN (Wet origin), Sterile pooled inoculate (10% Volume)	8.0	7.6	31.3	1.0	233	19
MN (Wet origin), Live pooled inoculate (10% Volume)	8.0	6.8	32.5	2.8	214	3
BACKGROUND Mix (90% Volume)	7.4	1.0	21.0	8.3	383	38

During the first 2 weeks of the experiment, plants received water every 1–2 days. Subsequently, we exposed plants to two watering treatments: wet (watered every 2–3 days) and dry (watered every 7 days) to field capacity. To minimize differences in nutrient availability due to soil sterilization (Table 1), and to ensure that plants were primarily limited by water, not nutrients, all plants received 20 ml of a 100 ppm 20N-2P-20K fertilizer on 28 July, 17 September and 3 October, 2016. All plants were destructively harvested on 18 Oct 2016. At this time we measured plant traits (described below) and also collected 10 mL of rhizoshere soil, which we sampled below the inoculum layer to ensure that we only characterized soil fungi that had proliferated during the experiment. We also collected a subset of fine roots for assessments of root colonization by AMF as well as other fungi (described below).

### Plant trait measurements

We measured total biomass production plus five plant functional traits. Three traits were related to resource acquisition stem height, root:shoot ratio, and specific leaf area (i.e., leaf area per unit leaf dry mass; SLA). The other two traits were latex production and trichome density. Latex is a sticky substance exuded from specialized canals that run throughout the aboveground plant tissues that primarily functions as defense against herbivores (Agrawal and Konno, 2009). Trichomes can function as a defense trait, but also as a drought tolerant trait (Agrawal and Fishbein, 2006; Agrawal et al., 2009).

To measure leaf traits we harvested one of the top fully expanded leaves from each plant. Harvested leaves were refrigerated for < 48 h and then scanned. Trichome density was counted under a dissecting scope in a 33 mm^2^ area on the lower surface of the leaf and then the leaves were dried at 60°C for 48 h. Specific leaf area (SLA) was calculated as the area (cm^2^) per unit mass (g). Immediately after individual leaves were harvested from each plant we captured exuded latex from the stem on a pre-weighed 1 cm diameter filter paper, which was then placed into a pre-weighed centrifuge tube. Centrifuge tubes were kept frozen and then weighed to the nearest 0.1 mg. Latex production was quantified as fresh weight (Agrawal and Fishbein, 2006; Waller et al., 2018). We then measured stem height on each plant from the soil to the apical meristem and harvested all above- and belowground biomass. Biomass was separated into above and belowground parts to allow calculation of root:shoot ratio, dried for at 60°C for 48 h, and then weighed.

### Fungal colonization of roots

Fine roots (< 1 mm diameter) were cleaned and stained in trypan blue (Phillips and Hayman, 1970; Brundrett et al., 1996) and fungal colonization was determined using the gridline intersect method based on approximately 50 intercepts per sample (McGonigle et al., 1990). Arbuscular mycorrhizal fungi were identified using morphological features associated with AMF, such as arbuscules, coils, vesicles and dichotmous branching patterns of mostly non-septate hyphae (Smith and Read, 2008). All other fungi (those staining blue as well as dark septate) not possessing these features were quantified as non-AMF (Figure S1). This approach remains the most commonly used method to assess AMF and parasitic fungal colonization (Smith and Read, 2008).

### Molecular characterization of fungal communities

#### DNA extraction and PCR

We collected 10 soil samples from 0 to 15 cm deep around an individual *A. speciosa* plant per site from 6 sites across the moisture gradient for a total of 60 field soil samples. At the end of the greenhouse experiment, soil samples were also collected from 15 pots per live soil treatment (two inocula × two watering treatments) for a total of 60 greenhouse samples. Field and greenhouse soil was freeze-dried using Labconco Freezone benchtop freeze dry system (Labconco, Kansas City, MO, United States). Genomic DNA was extracted from ~250 to 300 mg dried soil per sample using a PowerSoil™ DNA isolation kit (MoBio Laboratories, Inc., Solana Beach, CA, United States), following the manufacturer's instructions. Samples were then prepared for Illumina sequencing using a two-step PCR protocol to first amplify our target region and then attach unique sample identifiers. Detailed descriptions are in Bullington et al. (2018) and Lekberg et al. (2018). Briefly, the ITS2 region was amplified to target all fungi using general fungal primers, which included a mix of forward fungal primers flITS7 (Ihrmark et al., 2012) and flITS7o (Kohout et al., 2014) and the reverse primer ITS4 (White et al., 1990). Because general fungal ITS primers can sometimes result in poor amplification of AMF (Lekberg et al., 2018), we used the AMF-specific primers WANDA and AML2 (Lee et al., 2008; Dumbrell et al., 2011) targeting the small subunit (SSU) rRNA gene to characterize AMF communities. All PCR amplification was performed in a Techne TC-4000 thermocycler (Bibby Scientific, Burlington, NJ, United States). The second PCR reaction to attach sample-specific barcodes was the same for both SSU and ITS2 and followed Bullington et al. (2018). Resulting samples were pooled based on band intensities in a 1.5% agarose gel electrophoresis of PCR 2 product. Sequencing was done at the Institute for Bioinformatics and Evolutionary Studies (iBEST) genomics resources core at the University of Idaho (http://www.ibest.uidaho.edu/; Moscow, ID, United States). Amplicon libraries were sequenced using 2 × 300 paired-end reads on an Illumina MiSeq sequencing platform (Illumina Inc., San Diego, CA, United States).

#### Bioinformatics analysis

Initial bioinformatics analyses were conducted using “Quantitative insights into microbial ecology 2” (QIIME2 version 2017.12; https://qiime2.org/; Caporaso et al., 2010). Sequence reads were demultiplexed using the q2-demux plugin (https://github.com/qiime2/q2-demux). Forward and reverse reads were trimmed at 220 and 180 base pairs, respectively and paired for the ITS2 region only. Only forward reads were used for AMF, because the overlap between the forward and reverse reads is often too short to successfully merge the two without losing a lot of sequences. Restricting the AMF analyses to forward reads only should not influence our ability to identify AMF, because the forward read alone covers most of the highly variable region (Lee et al., 2008). Paired and unpaired sequences were quality filtered and de-replicated with the q2-dada2 plugin (Callahan et al., 2016), which simultaneously removes chimeras. The q2-dada2 plugin uses nucleotide quality scores to produce sequence variants (SVs), or sequence clusters with 100% similarity representing the estimated true biological variation within each sample. Although sequences are clustered at 100% similarity as opposed to the traditional 97% similarity, DADA2 produces fewer spurious sequences, fewer clusters, and results in a more accurate representation of the true biological variation present (Callahan et al., 2016). All SVs were assigned a taxonomic classification using the UNITE fungal ITS sequence database (Kõljalg et al., 2013) as a reference database for ITS2, and to a virtual taxon using MaarjAM (Öpik et al., 2010) as a reference database for AMF. The QIIME2 q2-feature-classifier (https://github.com/qiime2/q2-feature-classifier), a naive Bayes machine-learning classifier, which has been shown to meet or exceed classification accuracy of other existing methods (Bokulich et al., 2017), was used to assign taxonomy for ITS2 and SSU independently, using a confidence threshold of 0.94 as recommended for fungi in Bokulich et al. (2017). All non-fungal sequences were subsequently filtered out of each dataset before further analyses. Functional guild analysis of soil ITS2 data was performed according to Nguyen et al. (2016) using FUNGuild, which is an open access curated database that parses SVs into guilds based on taxonomic assignment. We focused this analysis on SVs that classified either as “AMF” or as “plant pathogens,” with either “probable” or “highly probable” confidence as stated in FUNGuild. We used shifts in sequence numbers in various treatments to assess potential shifts in relative abundance of the different guilds. It should be noted, however, that some fungi do not fall exclusively into a single guild, but may present as multiple guilds depending on resource availability and life stage (Nguyen et al., 2016) and many fungal ITS2 sequences present in this study were not assignable to any guild.

### Statistical analyses

#### Analysis of soil fungal communities

All statistical analyses associated with the fungal community composition in the field and greenhouse were conducted in R (R Core Team, 2017) using the vegan package (Oksanen et al., 2017) except where otherwise noted. All analyses were based on data rarefied to sequencing depth of 3300 for ITS2 data, 400 for field SSU data and 510 for greenhouse SSU data. These sampling depths were chosen based on saturation of species accumulation curves produced in QIIME2 (Figure S2). All samples were retained at these sequencing depths except in the SSU field data set where five samples were lost due to poor amplification. To assess if fungal community composition differed between the six sites and two regions or correlated with mean annual precipitation (field survey) or between the two pooled inocula (MN and WA) and watering treatments (greenhouse experiment), we performed permutation multivariate analyses of variance (Permanova) using the *adonis2* function in the vegan package in R with 999 permutations of Bray-Curtis distance matrices of Hellinger-transformed relative sequence abundance. For all field data analyses, site was used as a blocking factor nested within region. To visually assess patterns in soil fungal community composition between wet and dry regions in the field and treatments combination in the greenhouse, we used non-metric multidimensional scaling on the same distance matrices as the Permanova using the *metaMDS* function. NMDS results were plotted using the R package ggplot2 (Wickham, 2009). To compare richness (based on SVs) and relative sequence abundances of pathogens and AMF within our soil samples, we performed a two-way analysis of variance (ANOVA) using log or square-root transformed data where necessary to reduce variance heterogeneity. Correlations between pathogen and AMF richness and site-level precipitation were analyzed using Pearson's product-moment correlations.

#### Analysis of plant trait responses to soil biota

To ensure that all variables were comparable we centered all variables to a mean of zero and scaled their standard deviation to one. To improve normality, height and SLA were natural-log transformed and latex was square-root transformed prior to standardization. To evaluate the response of individual traits to treatments, we conducted multilevel model ANOVA. The multilevel model is conceptually similar to MANOVA and quantitatively similar to redundancy analysis (Jackson et al., 2012). We evaluated the soil inocula (i.e., live vs. sterile soil) explicitly in order to avoid inflating Type I errors and to facilitate the use of more robust statistical contrasts than would be possible through analyzing ratios (i.e., live:sterile soil, Rinella and Reinhart, 2018). The predictor variables in our models were plant trait (i.e., six levels), soil inoculum origin (MN or WA), watering treatment (wet or dry), soil biota treatment (live or sterile soil), and all possible interactions. Plant population was included as a random effect along with a term that included all experimental treatments nested within population to account for the multiple traits measured on each individual plant.

Of interest to our hypotheses were the soil biota × soil inoculum origin term, which tests the hypothesis that plant responses to soil biota depends on soil inoculum origin, and the biota × watering treatment, which tests for plasticity in how plants respond to soil biota. The trait × soil biota interaction term tests whether the six plant traits respond differently to soil biota. The three-way interactions, trait × soil biota × soil inoculum origin or trait × soil biota × watering treatment, would further indicate that the geographic or plastic responses to soil biota differ among plant traits. We do not focus on potential interactions between the experimental treatment and plant populations (i.e., testing whether plant populations respond differently to the treatments), because in a previous study we found no difference in responsiveness to AMF inoculations (Waller et al., 2018) and preliminary screening of our data showed no interactions with plant populations. We used *post-hoc* linear contrasts to evaluate how each trait responded to significant predictor variables and interaction terms.

We also tested whether fungal colonization of roots growing in live soil differed among the soil inoculum origin and watering treatments by quantifying percent colonization by AMF hyphae, arbuscules, and vesicles. We analyzed each of these separately, with fixed-effect predictor variables including soil inoculum origin (MN or WA), watering treatment (wet or dry), and their interaction. Plant population was included as a random effect. We also correlated percent AMF colonization with plant traits in treatments that were significantly affect by the microbial treatment in the dry and wet watering treatments.

Multilevel ANOVAs were run using the lmer function in the lme4 package (Bates et al., 2013). *F-* and *p*-values were estimated using the anova function in the lmerTest package, with the Satterthwaite approximation to estimate denominator degrees of freedom (Kuznetsova et al., 2016). *Post-hoc* contrasts were constructed using the lsmeans package in R (Lenth, 2013).

## Results

### Soil fungal communities in wet and dry regions

Targeting the whole fungal community, we recovered 5673 SVs from the six field sites compared to just 2393 SVs in greenhouse soil at the end of the experiment, with 456 SVs recovered from both greenhouse and field. SV turnover was higher in field samples than greenhouse samples, with only 21.4% of SVs found in more than 1 plant in the field compared to 42.0% in greenhouse samples. No SV was found in more than 25% of all field samples. Based on ITS2 sequences in UNITE, the most abundant fungal SVs in the field matched most closely to fungi in the genus *Mortierella* and unknown *Basidiomycota*, compared to *Chaetomium* (found in 75% of greenhouse samples) and *Spizellomyces* in the greenhouse.

Using site as a blocking factor, there were no differences in total fungal richness between wet (MN) and dry (WA) regions in the field (Table 2C). Richness did differ among individual sites (Table 2C), however, with the driest site in WA having lower fungal SV richness based on ITS2 data. Fungal community composition (all fungi, pathogens, and AMF) in the field differed between regions and sites (Table 2E) and was additionally related to mean annual precipitation (*F* = 1.5, *p* = 0.001, Figure S3).

**Table 2:** ANOVA tables for relative sequence abundance from the **(A)** field and **(B)** greenhouse; sequence variant (SV) richness in the **(C)** field and **(D)** greenhouse; and perMANOVA table for community composition in the **(E)** field and **(F)** greenhouse.

**(A) Field data-relative abundance**	***F***	***p***	**(B) Greenhouse data-relative abundance**	***F***	***p***
**Pathogens~Region/Site**	**6.2**	**0.016**	**Pathogens~Treatment**	**10.8**	**0.001**
**Pathogens~Site**	**9.5**	**0.001**	Pathogens~Inocula	1.4	0.258
AMF~Region	3.3	0.076	Pathogens~Treatment × Inocula	0.1	0.764
**AMF~Site**	**6.3**	**0.001**	**AMF~Treatment**	**57.2**	**0.001**
			AMF~Inocula	0.0	0.874
			AMF~Treatment × Inocula	2.9	0.094
**(C) Field data - SV richness**	***F***	***p***	**(D) Greenhouse data-SV richness**	***F***	***p***
All Fungi~Region/Site	1.1	0.290	All Fungi~Treatment	1.3	0.258
**All Fungi~Site**	**11. 3**	**0.001**	All Fungi~Inocula	0.01	0.894
Pathogen~Region/Site	0.2	0.627	All Fungi~Treatment × Inocula	2.4	0.126
Pathogens~Site	**6.8**	**0.001**	**Pathogens~Treatment**	**15.2**	**0.001**
AMF~Region/Site	3.2	0.081	**Pathogens~Inocula**	**13.8**	**0.001**
**AMF~Site**	**17.4**	**0.001**	Pathogens~Treatment × Inocula	0.7	0.396
			**AMF~Treatment**	**87.0**	**0.001**
			**AMF~Inocula**	**15. 4**	**0.001**
			AMF~Treatment × Inocula	0.6	0.438
**(E) Field data-composition**	***F***	***p***	**(F) Greenhouse data-composition**	***F***	***p***
**All Fungi~Region/Site**	**1.6**	**0.001**	**All Fungi~Treatment**	**1.4**	**0.021**
**All Fungi~Site**	**1.4**	**0.001**	**All Fungi~Inocula**	**5.8**	**0.001**
**Pathogens~Region/Site**	**1.7**	**0.001**	**All Fungi~Treatment** × **Inocula**	**1.2**	**0.048**
**Pathogens~Site**	**1.5**	**0.001**	Pathogens~Treatment	1.2	0.203
**AMF~Region/Site**	**10.2**	**0.001**	**Pathogens~Inocula**	**10.4**	**0.001**
**AMF~Site**	**6.6**	**0.001**	Pathogens~Treatment × Inocula	1.1	0.246
			**AMF~Treatment**	**5.350**	**0.001**
			**AMF~Inocula**	**9.498**	**0.001**
			**AMF~Treatment** × **Inocula**	**3.731**	**0.001**

*Significant terms (p < 0.05) are bolded*.

**Figure 2:**
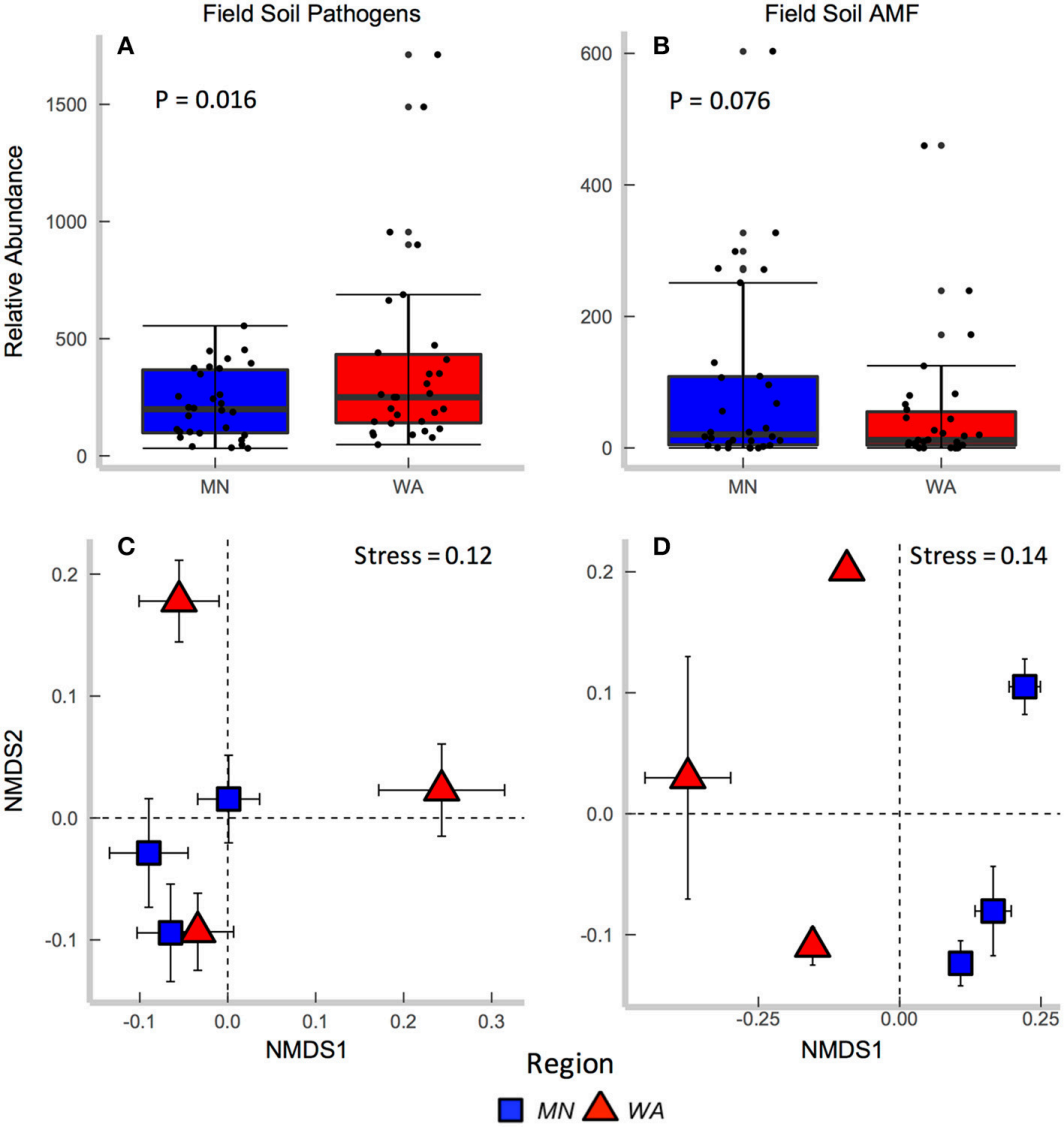
Relative abundance of **(A)** fungal pathogens and **(B)** AMF in field collected soil. Centroids of ordinations of soil fungi community compositions for **(C)** pathogens and **(D)** AMF.

According to FUNGuild, 8.2% of all ITS2 sequences in field soil were classified as “probable pathogens,” and this abundance was higher in the dry than in wet region (Figure 2A and Table S2). In contrast to our hypothesis, the highest abundance of pathogens was observed in the site with the least mean annual precipitation, and pathogen abundance correlated negatively with mean annual precipitation across sites (*R* = −0.31, *p* = 0.02). The composition (Table 2E), but not richness (Table 2C), of fungal pathogen communities differed between regions as well (Figure 2C) and was also related to mean annual precipitation (*F* = 1.6, *p* = 0.001).

AMF were represented by 1.9% of total ITS2 sequences (Table S3). Relative abundance (Table 2A) and richness (Table 2C) of AMF differed across sites but not region (Figure 2B), and both were highest in the site in North Dakota. AMF community composition (based on SSU sequence data) also varied between the two regions (Figure 2D) and across all sites (Table 2E) and additionally related to mean annual precipitation (*F* = 6.3, *p* = 0.001).

### Greenhouse experiment

#### Fungal community differences between the MN and WA pooled inocula and responses to soil moisture

**Figure 3:**
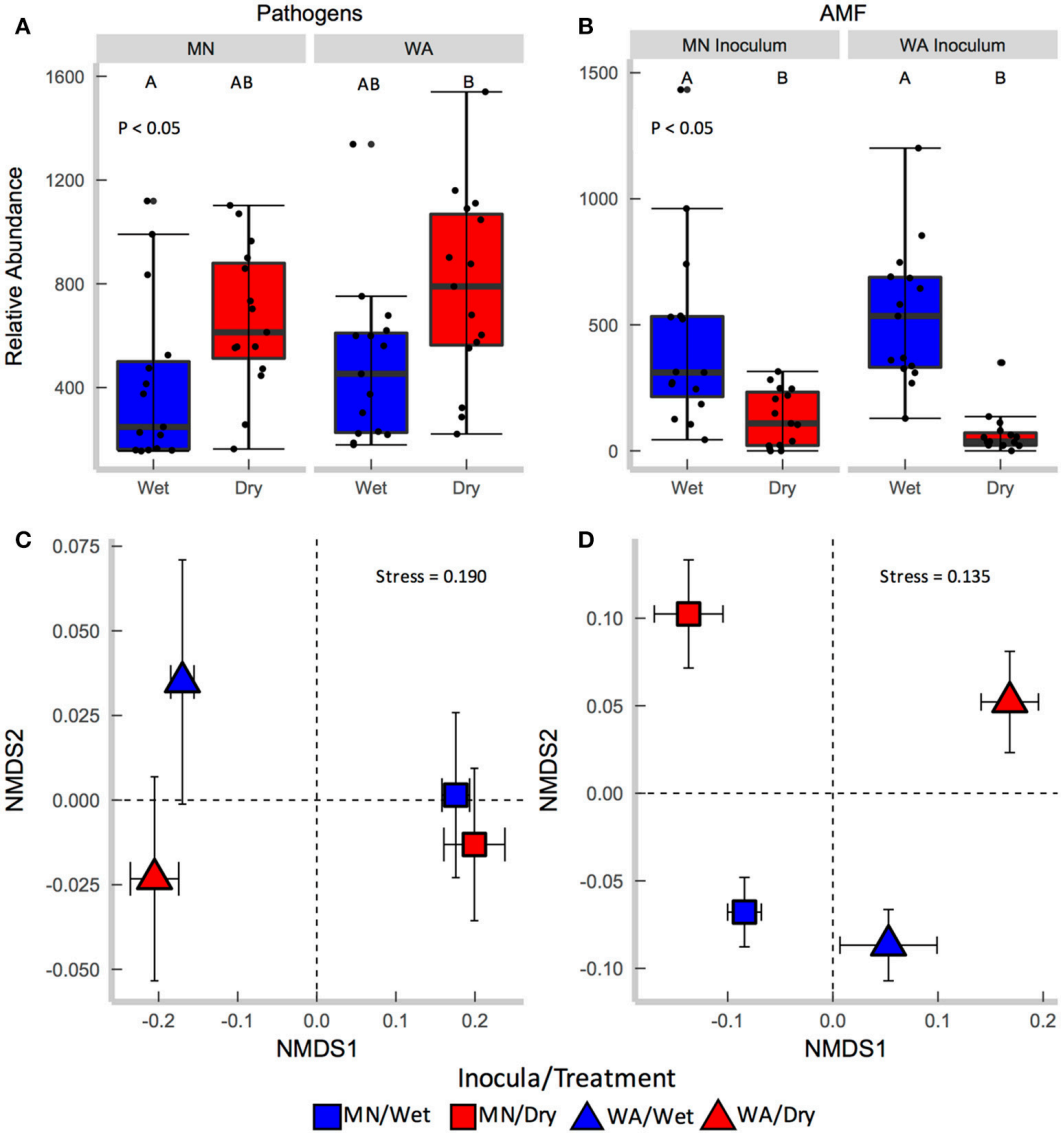
Relative abundance of **(A)** fungal pathogens and **(B)** AMF in soil collected after the greenhouse experiment. Centroids of ordinations of soil fungi community compositions for **(C)** pathogens and **(D)** AMF.

Each source of pooled inocula (WA and MN) had a higher relative abundance of pathogens in dry than in wet soils (Table 2B and Figure 3A), whereas AMF showed the opposite pattern and were more abundant in wet than dry soil (Figure 3B). Overall fungal richness did not differ between the WA and MN inocula or the two watering treatments (Table 2D), but composition did (Table 2F). Pathogens were represented by 17.3% of fungal sequences in the greenhouse. According to FUNGuild, 66% of pathogen sequences matched most closely to the genus *Spizellomyces*. AMF made up 8.7% of ITS2 sequences. The richness of pathogens and AMF was higher in soils inoculated with WA inoculum than MN inoculum, and was higher for pathogens in dry soil and higher for AMF in wet soils (Table 2D). The composition of pathogens differed between inocula, but not between moisture treatments (Figure 3C). AMF composition on the other hand, differed between both soil inocula and watering treatments (Figure 3D), but all communities tended to be dominated by Glomeraceae and Claroideoglomeraceae AMF (Hahn et al., 2018). For AMF, the extent of shift due to watering treatment depended on the inoculum source (Table 2F and Figure 3D).

#### AMF and non-AMF root colonization

**Figure 4:**
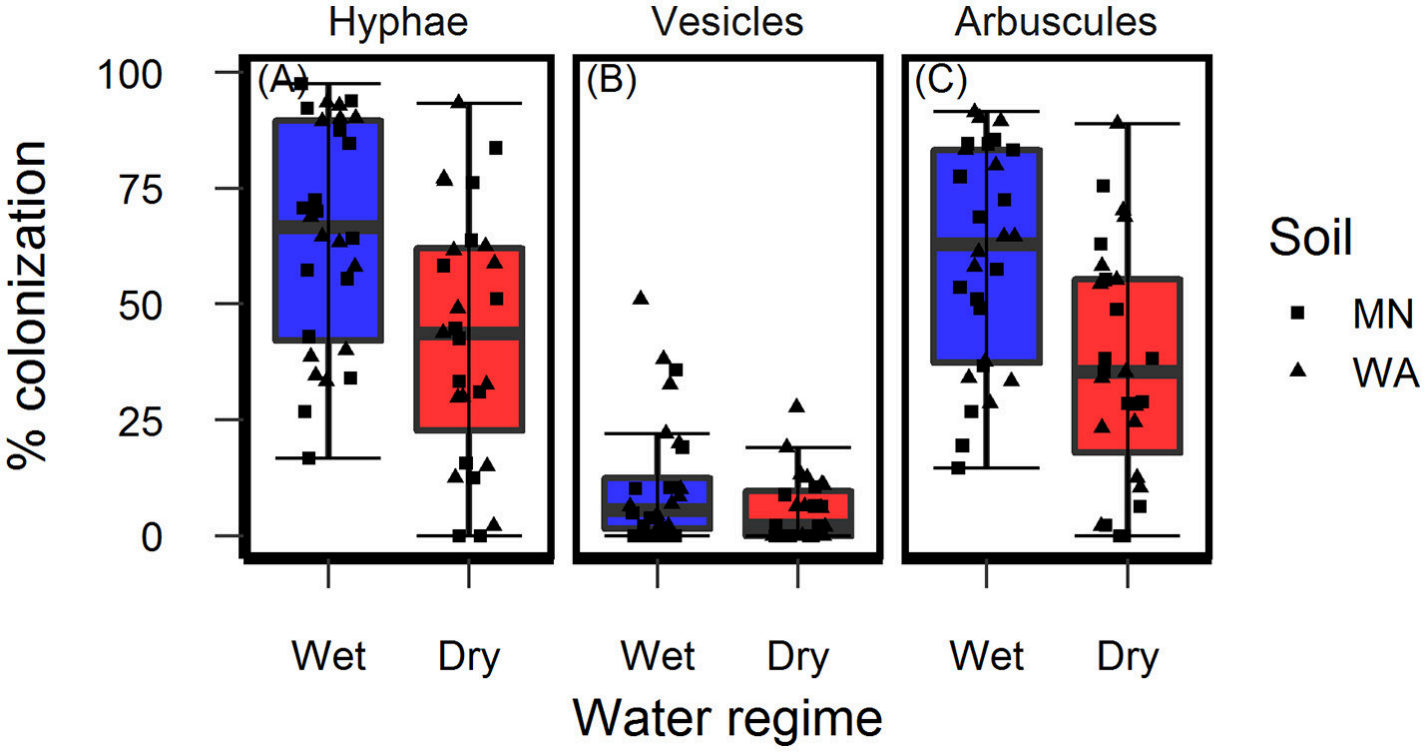
Colonization of AMF **(A)** hyphae, **(B)** vesicles, and **(C)** arbuscules on *Asclepias speciosa* plants growing in live soil exposed to dry and wet watering treatments.

Colonization of roots by AMF hyphae and arbuscules was affected by the watering treatment [hyphae: *F*_(1, 49.3)_ = 10.4, *p* = 0.002 and arbuscules: *F*_(1, 49.3)_ = 13.0, *P* < 0.001] and was higher in wet than dry soil (Figures 4A,C) regardless of soil biota origin. Percent colonization by vesicles was affected by the watering treatment [*F*_(1, 49.2)_ = 4.2, *P* = 0.043] and soil inoculum origin [*F*_(1, 49.3)_ = 7.7, *P* = 0.008], and were three-times more abundant in the pooled WA soil inoculum (mean = 12.0%, *se* = 2.3) than the pooled MN inoculum (mean = 4.5%, *se* = 2.3, Figure 4B). The colonization by fungi other than AMF was low across all treatments (1.6% ± 0.36, mean ± se) and there were no effects of either soil biota origin or watering treatment.

#### Plant responses

**Figure 5:**
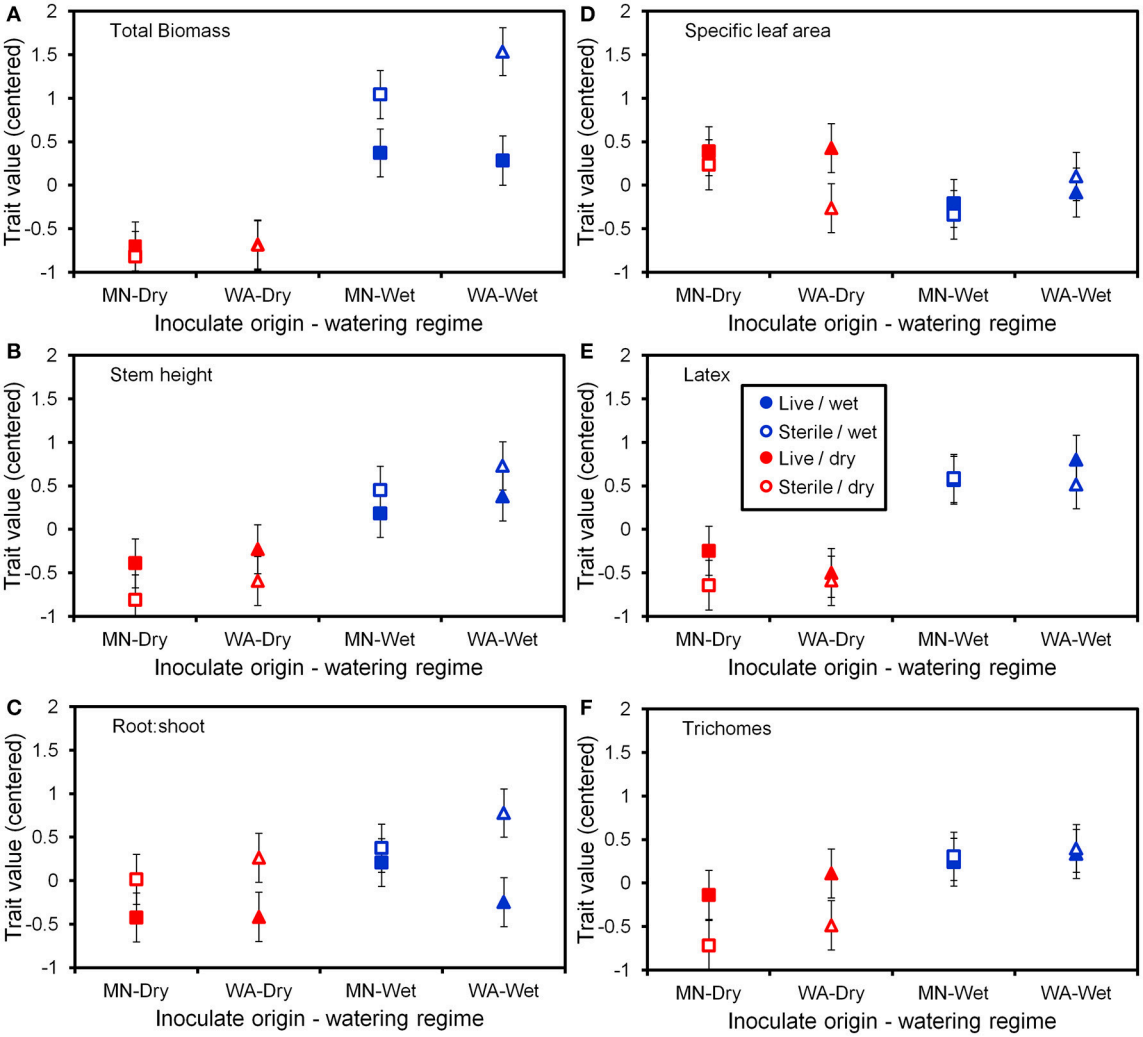
Trait values (centered) for the six plant functional traits **(A–F)** measured at the end of the greenhouse experiment. Soil inoculates originated from wet field sites (MN) or dry field sites (WA). Bars are ±1 SE. Square symbols indicate soil inoculate originating from MN and triangles indicate soil inoculate originating from WA.

The two soil inocula (WA or MN) did not differ in their influence on plant responses and did not statistically interact with any other term (Table 3). The main effect of the watering treatment was highly significant (Table 3), with most traits increasing in wet vs. dry soils (Figure 5). The two-way interaction between soil biota treatment (i.e., live or sterile treatments regardless of soil biota origin) and watering treatment was significant (Table 3). Comparing the trait responses to zero in each of the watering treatments, the average trait value (averaged across all plant traits) response to soil biota in the dry treatment was marginally positive (linear contrast: live-sterile in dry = 0.19, *se* = 0.11, *df* = 188.5, *t* = 1.7, *p* = 0.090), whereas the average trait value response in the wet treatment was significantly negative (linear contrast: live-sterile in wet = −0.30, *se* = 0.11, *df* = 186.8, *t* = −2.75, *p* = 0.007). Comparing the magnitude of trait responses between the dry and wet treatments, the effect of the soil biota treatment on plant traits (averaged across all six traits) was significantly more positive in the dry watering treatment compared to the wet treatment (linear contrast on [live-sterile in dry, estimate = 0.19]-[live-sterile in wet, estimate = −0.30] averaged across all plant traits = 0.50, *se* = 0.16, *df* = 187.6, *t* = 3.14, *p* = 0.002). There was also a significant two-way interaction between plant traits and soil microbes (Table 3), suggesting the traits responded differently to live vs. sterile soil. There was also a significant two-interaction between plant traits and watering treatment (Table 3), suggesting that the plant traits responded differently to the watering treatment.

**Table 3:** ANOVA table from the multilevel model of plant traits from the greenhouse experiment.

**Effect**	**DF**	***F***	***p***
Trait	5, 187.43	0.16	0.977
Soil inoculum origin	1, 187.55	1.54	0.216
Soil biota (live vs. sterile)	1, 187.74	0.50	0.480
**Watering regime** (dry or wet)	**1, 188.38**	**82.40**	<**0.001**
Trait × Inoculum	5, 187.43	0.21	0.958
**Trait**×**Biota**	5, 187.43	**3.53**	**0.005**
Inoculum × Biota	1, 187.54	0.79	0.374
**Trait**×**Water**	**5, 187.43**	**11.04**	<**0.001**
Inoculum × Water	1, 187.62	0.37	0.542
**Biota**×**Water**	**1, 187.54**	**9.87**	**0.002**
Trait × Inoculum × Biota	5, 187.43	0.42	0.831
Trait × Inoculum × Water	5, 187.43	0.41	0.840
Trait × Biota × Water	5, 187.43	0.95	0.449
Inoculum × Biota × Water	2, 187.57	0.53	0.469
Trait × Inoculum × Biota × Water	5, 187.43	0.47	0.800
^†^Plant population		3.34	0.070
^†^**Nested term**		**159.38**	<**0.001**

*Significant terms (p < 0.05) are bolded*.

† *Random effects*;

*χ^2^ values are shown in F column*.

**Figure 6:**
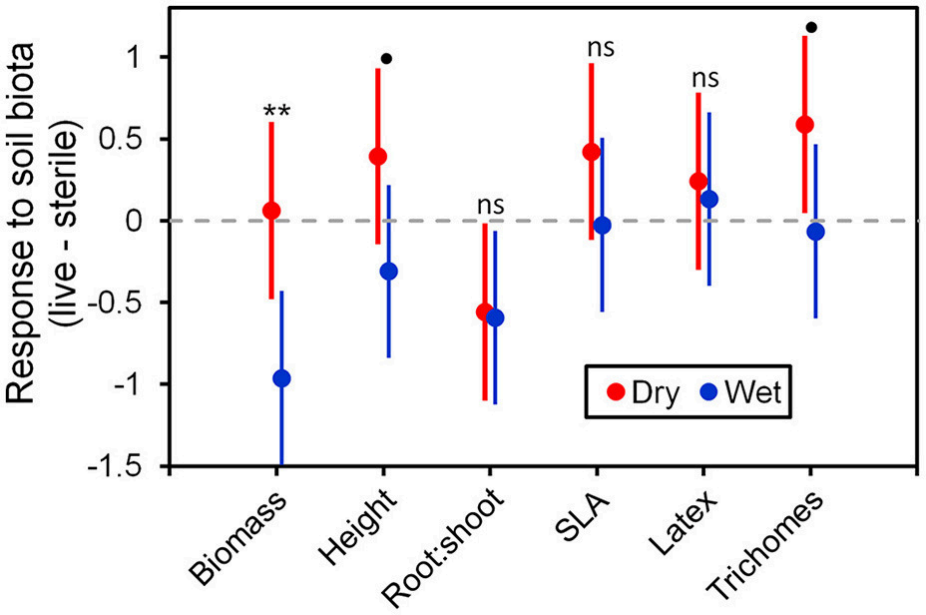
Response to soil microbes, contrasts estimated as least-square means in live minus sterilized soil, for the six measured traits under wet and dry watering regimes. Bars are 95% confidence intervals. Error bars that do not overlap zero indicate that that response differed from zero (i.e., response ≠ 0). Different responses between wet and dry watering treatment are indicated as follows: **P < 0.01; ^•^*P* < 0.1.

To more fully understand how the individual plant traits responded to soil biota in wet vs. dry watering treatments, we performed two types of (*a priori*) linear contrasts specifically related to our hypotheses. First, we constructed contrasts to compare whether the response of soil biota for each trait (i.e., trait value in live-sterile) was significantly different than zero in each of the watering treatments. In the dry treatment, root:shoot ratio responded negatively to soil biota and trichomes responded positively to soil biota (Figure 6 and Table 4). No other traits were significantly affected by soil biota in the dry treatment (Figure 6 and Table 4). In the wet treatment, biomass and root:shoot ratio both responded negatively to soil biota (Figure 6 and Table 4). No other traits were significantly affected by soil biota in the wet treatment. Second, we used linear contrasts to compare (the trait value for live-sterile in dry)-(the trait value for live-sterile in wet). The linear contrasts for biomass in live-sterile were significantly different (Figure 6, Table 4). The contrasts for height and trichomes were marginally different (Figure 6 and Table 4). Contrasts for the other traits did not differ (Figure 6 and Table 4).

**Table 4:** Contrasts from the multilevel mixed model from the greenhouse experiment.

**Trait**	**Contrast**	**Estimate**	**SE**	**df**	***t***	***P***
Biomass	Live–Sterile in “dry”	0.063	0.276	188.7	0.23	0.8206
Height	Live–Sterile in “dry”	0.394	0.275	186.1	1.43	0.1539
**Root:shoot**	**Live–Sterile in “dry”**	−**0.558**	**0.276**	**188.7**	−**2.02**	**0.0448**
SLA	Live–Sterile in “dry”	0.423	0.276	188.7	1.53	0.1271
Latex	Live–Sterile in “dry”	0.242	0.276	188.7	0.88	0.3828
**Trichomes**	**Live–Sterile in “dry”**	**0.589**	**0.276**	**189.7**	**2.13**	**0.0345**
**Biomass**	**Live–Sterile in “wet”**	−**0.962**	**0.271**	**187.2**	−**3.55**	**0.0005**
Height	Live–Sterile in “wet”	**–**0.309	0.270	183.2	**–**1.15	0.2530
**Root:shoot**	**Live–Sterile in “wet”**	−**0.594**	**0.271**	**187.2**	−**2.19**	**0.0296**
SLA	Live–Sterile in “wet”	**–**0.026	0.271	187.2	**–**0.10	0.9230
Latex	Live–Sterile in “wet”	0.133	0.271	187.2	0.49	0.6233
Trichomes	Live–Sterile in “wet”	**–**0.065	0.271	187.2	**–**0.24	0.8109
**Biomass**	**(LiveDry–SterileDry) – (LiveWet–SterileWet)**	**1.024**	**0.387**	**187.9**	**2.65**	**0.0088**
*Height*	*(LiveDry –SterileDry)–(LiveWet-SterileWet)*	*0.703*	*0.385*	*184.7*	*1.83*	*0.0696*
Root:shoot	(LiveDry –SterileDry)–(LiveWet–SterileWet)	0.036	0.387	187.9	0.09	0.9265
SLA	(LiveDry–SterileDry)–(LiveWet–SterileWet)	0.449	0.387	187.9	1.16	0.2467
Latex	(LiveDry –SterileDry) –(LiveWet–SterileWet)	0.108	0.387	187.9	0.28	0.7797
*Trichomes*	*(LiveDry –SterileDry)–(LiveWet-SterileWet)*	*0.653*	*0.387*	*188.4*	*1.69*	*0.0929*

*Significant contrasts are bolded (P < 0.05) and marginally significant contrasts are italicized (P < 0.1)*.

## Discussion

Our goal was to test how soil resource levels influenced soil fungal communities and plant responses to these communities. We were also interested in understanding whether soil biota and plant responses differed depending on soil biota origin across a resource gradient in the field. We show that AMF and fungal pathogen communities differed broadly between geographic regions that differ in precipitation. However, functionally we observed no difference in how plants responded to pooled inoculum from each region in the greenhouse. Thus, while long-term environmental conditions could have contributed to the regional differences in fungal communities we observed, these disparate fungal communities possessed high functional redundancy. Plant responses were more strongly driven by short-term resource availability, but the extent and direction of these responses depended on the specific plant trait. This highlights the complex relationships between resource availability and the outcome of plant-soil biota interactions.

### Resource supply drives trait-specific responses to soil biota

Water additions increased plant biomass, indicating that plants were resource limited in dry soils, either by water directly or via soil moisture-mediated effects on nutrient availability. Based on findings from work along fertility gradients (Johnson, 1993; Johnson et al., 1997; Leff et al., 2015), we predicted that soil biota would be beneficial when resources where limiting and detrimental when resources were abundant. Our results indicate that these relationships also apply along soil moisture gradients, because plant responses were neutral to positive under drought conditions, but negative in well-watered soil (Figure 6). This conditional response was especially strong for plant biomass; plants did not respond to soil biota in live dry soil, but responded negatively in live wet soil (Figure 5). In contrast, soil biota also influenced trichome density, but only in dry soil (Figure 6). This is perhaps not surprising since trichomes can increase drought tolerance (Farquhar and Richards, 1984; Agrawal et al., 2009) in addition to enhancing herbivore defense (Agrawal and Fishbein, 2006). We do not know which component of soil biota caused this effect, but inoculations with AMF alone have increased trichome density in a previous study (Waller et al., 2018) and all plants were highly colonized by AMF in our study (Figure 4). Given this high root colonization, the neutral or even negative plant responses to live soil were surprising, especially because *A. speciosa* and other *Asclepias* species generally benefit from AMF inoculations (Wilson and Hartnett, 1998; Busby et al., 2011; Tao et al., 2016; Waller et al., 2018).

One possible explanation for the above patterns is that there are negative correlations in the responsiveness of multiple traits, particularly between biomass and trichomes (Waller et al., 2018), or other unmeasured traits such as plant secondary metabolites (e.g., cardenolides; Vannette et al., 2013). Plants under dry conditions may have preferentially allocated resources to traits (i.e., trichomes) and soil biota that allow them to best cope with drought stress, which may not result in differences in biomass. It is also possible that strong, positive growth responses from AMF only occur when plants are limited by phosphorus (Smith and Read, 2008 and references therein), which, due to the high availability of this nutrient and repeated fertilizations (Table 1), was unlikely in this experiment. Interestingly, however, plants grown in live soil allocated less biomass to roots than plants grown in sterile soil, irrespective of watering treatment and soil biota origin (Figure 5C). Roots were also heavily colonized by arbuscules (Figure 4), which is where AMF deliver phosphorus to plants. Thus, it is possible that even though AMF did not promote growth and plants were not phosphorous-limited, fungal colonization prompted a shift in allocation patterns whereby AMF substituted for some root functions. Other work has found that AMF can be functionally important even in cases where growth is not affected (Smith, 2003). Alternatively, soil moisture may have shifted bacterial communities or function, which has been shown to influence plant performance (e.g., Letourneau et al., 2018). This study does not allow us to identify the underlying mechanisms of observed patterns. As such, measuring AMF-mediated phosphorous uptake, water use efficiency, shifts in allocation to various plant traits, or bacterial communities would be a productive future direction.

### Plant responses driven by shifts within rather than between fungal guilds

We predicted that plant growth responses to soil biota would be associated with the functional identity of the soil community. In other words, positive plant responses to soil biota should be associated with high mutualist to pathogen ratios whereas negative plant responses to soil biota should be associated with smaller mutualist to pathogen ratios. However, we found that root colonization by fungi other than AMF (which could include pathogens) was very low in all treatments. Furthermore, soil pathogen abundance (based on sequence abundance) was actually *higher* in dry than wet soil, which is inconsistent with the *less* negative plant responses in this treatment. The higher pathogen abundance in dry soil was unexpected, although some pathogens appear to thrive in dry soils (Cook and Papendick, 1972). Dry conditions could filter for fungi able to best tolerate desiccation and then reproduce quickly when wetted. It is also possible that these fungi experienced competitive release as a result of the lower AMF abundance (Borowicz, 2001), or that fungi classified as pathogens based on a match to the curated database FunGuild (Nguyen et al., 2016) may also function as saprotrophs. For example, *Fusarium*, which was recorded in our sequence data, is typically pathogenic to a narrow taxonomic host range, but saprotrophic strains can be broadly distributed across cultivated and native grassland soils (Gordon and Okamoto, 1989; Lozupone and Klein, 2002).

A more likely explanation for plant growth reductions in live, wet soil is that AMF were parasitic. AMF commonly function along a continuum from parasitism to mutualism (Johnson et al., 1997), and plant species, such as milkweed, that are very responsive to AMF under resource limiting conditions (Wilson and Hartnett, 1998; Busby et al., 2011) may also be more susceptible to parasitism when resources are not limiting (Grman, 2012). Indeed, AMF abundance in both roots and soil was higher in wet than dry soil (Figure 4), a pattern that has been documented previously (Bell et al., 2014). This greater fungal biomass could have imposed an excessive carbon drain where the cost of associating with AMF exceeded the benefits derived under these particular conditions.

### High functional redundancy among disparate fungal communities

We predicted that the origin of the soil inoculum would influence plant responses, such that soil biota sourced from wetter regions should have a stronger negative effect on plants when grown in wet soil than soil inoculum sourced from drier regions. This was not supported. Despite clear differences in both AMF and pathogen compositions in the field as well as in the pooled inocula in the greenhouse (Figures 2, 3), these soils had similar effects on plants (Figure 5 and Table 3). Because we pooled our inoculum from the gradient end points, we can only discuss the specific function of the pooled inocula, rather than making broad generalizations regarding regional differences in function. This pooling can also inflate Type I errors, i.e., falsely detecting statistically significant effects of soil inoculum origin (Reinhart and Rinella, 2016; Gundale et al., 2017; see discussions in Cahill et al., 2017). However, even with our liberal test, we found no apparent functional difference between the two soil inocula, despite clear differences in composition. This contrasts with some previous studies that have shown that soils experiencing drought can influence plant responses (Lau and Lennon, 2012; Kaisermann et al., 2017). However, while Kaisermann et al. (2017) found that soil microbial communities previously exposed to drought were less beneficial than those that had not experienced drought, Lau and Lennon (2012) showed that plants did better under drought when matched with a microbial community that had previously experienced drought. What explains these different results are unclear, but may be related to the degree, nature and duration of stress imposed in various studies (Hawkes et al., 2011; Evans and Wallenstein, 2012). For example, the range of water availability experienced by plants in our experiment may not have been outside the natural variation that these fungi experience across seasons, or due to extreme weather events. Seasonal differences in microbial communities may exceed effects of severe experimental reductions in precipitation (Cregger et al., 2012). Natural variation in rainfall may result in a “storage effect” where fungi with wide environmental tolerances coexist and where subsets are favored based on current environmental conditions (Hawkes et al., 2011).

The clear changes in AMF and pathogen communities in soil inocula experiencing drought indicate that soil moisture, or moisture-mediated shifts in either host plant status or nutrient availability can strongly influence fungal communities. Our results generally agree with previous studies that have shown shifts in fungal communities along precipitation gradients (Kivlin et al., 2011; Tedersoo et al., 2014; Zhang et al., 2016) and where soil moisture has been experimentally altered (Furze et al., 2017; Kaisermann et al., 2017; Meisner et al., 2018; Schmidt et al., 2018; She et al., 2018). These changes in composition may or may not result in altered fungal richness, which sometimes declines (Toberman et al., 2008; Gehring et al., 2017), show no difference (Schmidt et al., 2018; She et al., 2018), or even increase (Hawkes et al., 2011) with drought. We observed no regional difference in AMF richness, but a reduction in dry soils in the greenhouse, whereas pathogen richness was higher in the dry region and increased with experimental drought. Shifts in fungal richness can have functional consequences for plant growth and fitness as well as ecosystem processes (van der Heijden et al., 1998; Toberman et al., 2008; Lau and Lennon, 2011), but results to date suggest that responses to drought differ among studies and possibly fungal guilds.

### Limitations and future directions

In this study, we collected soils from the endpoints of a precipitation gradient in order to understand how fungal communities might be influenced by differences in soil moisture. However, soil nutrient availabilities tended to be higher in drier sites (Table S1) and other factors that differ among sites might drive fungal community differences instead or in addition to soil moisture. As well, the limited overlap in fungal communities among sites within regions is consistent with taxa being dispersal limited, which could further obscure filtering based on soil moisture (Cottenie, 2005; Lekberg et al., 2007; Vellend, 2010). These factors could help explain the lack of functional differences observed between the two pooled inocula in the greenhouse. However, regardless of what shaped these communities in the field, our study shows that disparate fungal communities respond in similar ways to short-term differences in soil moisture and have high functional redundancy.

Whether or not responses observed in the greenhouse would also occur in the field is uncertain however, because greenhouse conditions tend to favor disturbance tolerant soil biota that may not be abundant in the field. Similar to previous work that has quantified this so-called “cultivation bias” effect (Sýkorová et al., 2007; Schmidt et al., 2018), we observed little overlap between field and greenhouse communities (Figure S1, Hahn et al., 2018). This reinforces the need to conduct experiments in the field, wherever possible (Lekberg and Helgason, 2018), because greenhouse experiments may poorly predict field responses (Heinze et al., 2016). For example, while AMF are not infrequently parasitic in the greenhouse (e.g., Klironomos, 2003), does this also happen under more natural conditions in the field? If so, it could have very important consequences for how we understand their role in structuring plant communities.

### Summary

By linking changes in resource levels to shifts in composition and function of soil fungal communities, we show that distinct fungal communities that originate from disparate environments have similar directional responses and cause equivalent plant functional responses to short-term alterations in soil moisture. Overall, we found that plant trait responses to soil biota shifted from negative to neutral or slightly positive with declining resources (i.e., soil moisture) regardless of soil biota origin. Contrary to our predictions, however, the changes in plant responses were not driven by a shift *between* fungal guilds but rather *within* guilds. Furthermore, it is most likely AMF became parasitic in high-resource environments. Whether or not this also happens in the field is uncertain. Much could be learned from additional studies that jointly quantify how variation in resource availability in the field influences soil biota, and how this in-term affects plant responses.

## Data availability

The data will be made publically available via figshare at the time of publication (referenced as Hahn et al., 2018, doi: 10.6084/m9.figshare.5926378). Representative sequences from both target regions were submitted to GenBank and assigned the following accession numbers: ITS greenhouse: MH450315-MH452715; ITS Field: KBZH01000000; SSU greenhouse: MH453115-MH453369; SSU Field: MH452716-MH453114.

## Author contributions

PH, JM, BL, and YL conceived the experiments. All authors contributed to designing the experiments and collecting data. PH analyzed plant and AMF data. LB analyzed molecular data. All authors contributed to interpreting the data. PH, JM, LB, and YL wrote the manuscript with input from the other co-authors.

### Conflict of interest statement

The authors declare that the research was conducted in the absence of any commercial or financial relationships that could be construed as a potential conflict of interest.
