# Corrigendum: Effects of Short- and Long-Term Variation in Resource Conditions on Soil Fungal Communities and Plant Responses to Soil Biota

**DOI:** 10.3389/fpls.2018.01937

**Published:** 2019-01-07

**Authors:** Philip G. Hahn, Lorinda Bullington, Beau Larkin, Kelly LaFlamme, John L. Maron, Ylva Lekberg

**Affiliations:** ^1^Division of Biological Sciences, University of Montana, Missoula, MT, United States; ^2^MPG Ranch, Missoula, MT, United States; ^3^Department of Ecosystem and Conservation Sciences, University of Montana, Missoula, MT, United States

**Keywords:** arbuscular mycorrhizal fungi, context-dependent, drought stress, intraspecific variation, plant-soil feedback, plant defense, plant traits, soil fungi

In the original article, there was a mistake in Figure 4 as published. The labels on the x-axis were switched around and did not properly align with the plotted data. The corrected Figure 4 appears below.

**Figure 4:**
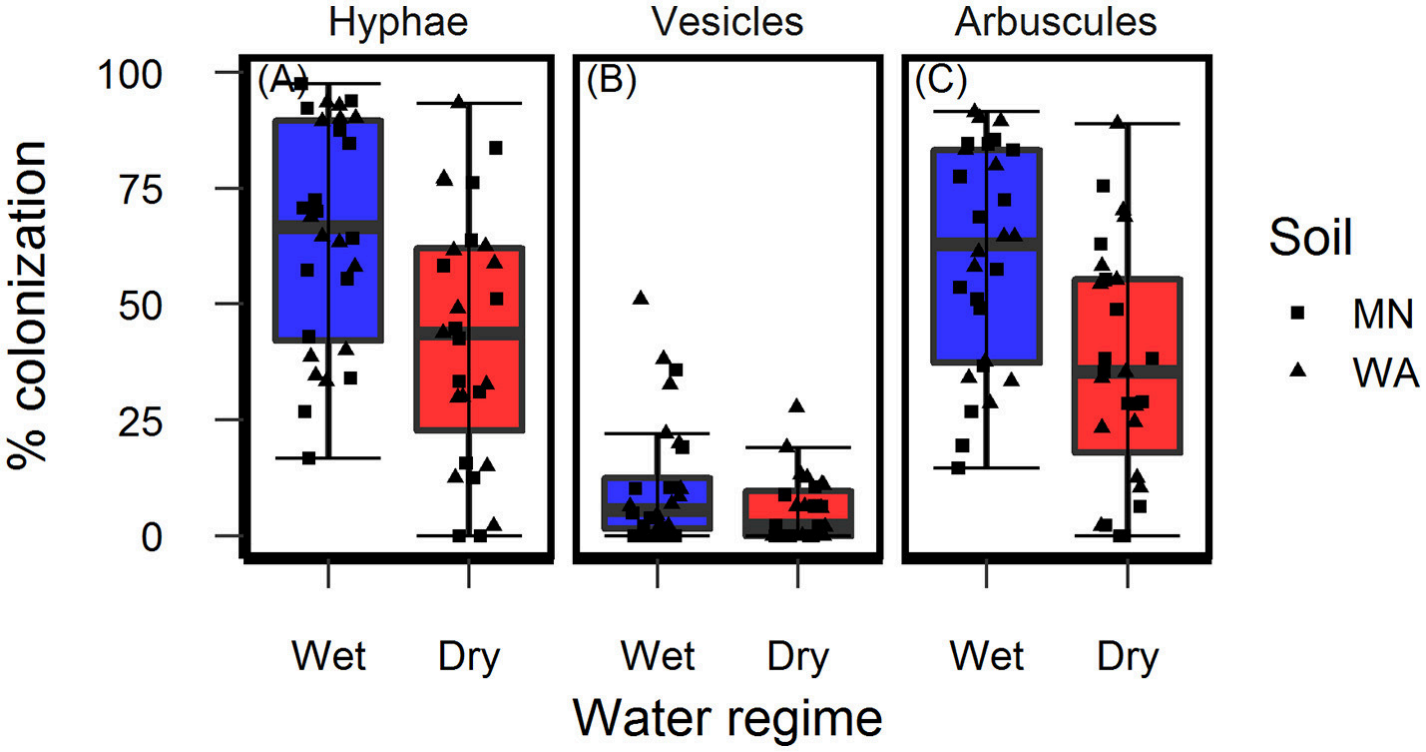
Colonization of AMF **(A)** hyphae, **(B)** vesicles, and **(C)** arbuscules on *Asclepias speciosa* plants growing in live soil exposed to dry and wet watering treatments.

The authors apologize for this error and state that this does not change the scientific conclusions of the article in any way. The original article has been updated.

