# Supplementary material for: Effects of Short- and Long-Term Variation in Resource Conditions on Soil Fungal Communities and Plant Responses to Soil Biota
Source: Front Plant Sci. 2018 Nov 6;9:1605. doi: 10.3389/fpls.2018.01605 (PMC6233719; doi:10.3389/fpls.2018.01605)
Supplement: Supplementary file 1 [file Table_1.DOCX]

**SUPPLEMENTAL MATERIALS FOR:**

**Effects of short- and long-term variation in resource conditions on soil fungal communities and plant responses to soil biota**

Philip G Hahn^1*^, Lorinda Bullington^2^, Beau Larkin^2^, Kelly LaFlamme^2^, John L Maron^1^, Ylva Lekberg^2,3^

1. Division of Biological Sciences, University of Montana, Missoula, MT, USA 59812

2. MPG Ranch, Missoula, MT, USA 59803

3. Department of Ecosystem and Conservation Sciences, University of Montana, Missoula, MT, USA 59812

* Correspondence: phil.hahn@mso.umt.edu

Running Title: Context dependency of plant-soil biota interactions

**List of supplemental materials:**

Table S1. Locations and climate information (30-year averages) for the study sites. Soils were pooled by region for the greenhouse experiment.

Table S2. List of most common arbuscular mycorrhizal and putative pathogen species found in the field soils. See data for a full list of sequence variants (doi: 10.6084/m9.figshare.5926378).

Figure S1. Roots harboring structures typical of (a) arbuscular mycorhizal fungi or (b) non-mycorrhizal fungi referred to as dark septate endophytes that do not absorb the trypan blue stain and remain brown.

Figure S2. Sequence variant accumulation curves for (a) SSU and (b) ITS regions. Each line represents a sample.

Figure S3. NMDS ordination of soil fungal communities based on sequence data from the field sites and greenhouse pots. Stress = 0.16. Field sites (circles) are coded by mean annual precipitation. Greenhouse pots (triangles) are coded by watering treatment.

Table S1. Locations and climate information (30-year averages) for the study sites. Soils were pooled by region for the greenhouse experiment.

| **Site code** | **Latitude** | **Longitude** | **Collected materials** | **MAT (°C)** | **MAP (mm)** | **Summer precip (mm)** | **pH** | **% OM** | **CEC** | **N** | **P** | **K** |
| --- | --- | --- | --- | --- | --- | --- | --- | --- | --- | --- | --- | --- |
| Mas | 43.90164 | -96.05658 | Soil only | 6.7 | 668 | 269 | 7.8 | 12.3 | 40 | 3.2 | 7 | 180 |
| Plo | 45.19875 | -96.25313 | Soil only | 6.7 | 585 | 246 | 7.8 | 14.7 | 37.8 | 0.7 | 5 | 188 |
| Tap | 46.88187 | -99.63670 | Both | 5.1 | 429 | 195 | 7.9 | 2.4 | 23.6 | 0.3 | 3 | 232 |
| Mng | 46.89105 | -103.38400 | Plants only | 5.7 | 400 | 177 | 8 | 4.5 | 22.2 | 0.8 | 6 | 204 |
| Ham | 46.22593 | -114.09644 | Both | 6.9 | 352 | 98 | NA | NA | NA | NA | NA | NA |
| Her | 46.90152 | -119.20535 | Both | 10.6 | 188 | 26 | 8.5 | 2.1 | 18.2 | 8.1 | 54 | 1115 |
| Ell | 47.00132 | -120.36391 | Both | 8.2 | 242 | 34 | 6.9 | 4.5 | 15.8 | 1.9 | 28 | 264 |

Table S2. List of 10 most abundant fungal pathogen taxa found in at least three samples in the field-collected soil samples of the MN and WA regions. For full species lists, refer to the data online (doi: 10.6084/m9.figshare.5926378).

| **State** | **Phylum** | **Class** | **Order** | **Family** | **Genus** | **Specific epithet** |
| --- | --- | --- | --- | --- | --- | --- |
| MN | Ascomycota | Sordariomycetes | Hypocreales | Nectriaceae | Fusarium | Solani |
| MN | Ascomycota | Dothideomycetes | Pleosporales | Pleosporaceae | Alternaria | Alternata |
| MN | Ascomycota | Sordariomycetes | Glomerellales | Plectosphaerellaceae | Plectosphaerella | Cucumerina |
| MN | Ascomycota | Sordariomycetes | Hypocreales | Nectriaceae | Fusarium | Oxysporum |
| MN | Chytridiomycota | Chytridiomycetes | Chytridiales | Chytridiaceae | Phlyctochytrium | Reinboldtiae |
| MN | Ascomycota | Sordariomycetes | Glomerellales | Plectosphaerellaceae | Gibellulopsis |  |
| MN | Ascomycota | Sordariomycetes | Xylariales | Microdochiaceae | Microdochium |  |
| MN | Ascomycota | Sordariomycetes | Hypocreales | Bionectriaceae | Clonostachys | Rosea |
| MN | Chytridiomycota | Spizellomycetes | Spizellomycetales | Spizellomycetaceae | Spizellomyces | Lactosolyticus |
| MN | Ascomycota | Sordariomycetes | Hypocreales | Nectriaceae | Fusarium |  |
| WA | Ascomycota | Sordariomycetes | Hypocreales | Nectriaceae | Fusarium | Solani |
| WA | Ascomycota | Sordariomycetes | Glomerellales | Plectosphaerellaceae | Plectosphaerella | Cucumerina |
| WA | Ascomycota | Dothideomycetes | Pleosporales | Didymellaceae |  |  |
| WA | Ascomycota | Dothideomycetes | Pleosporales | Pleosporaceae | Alternaria | Alternata |
| WA | Basidiomycota | Exobasidiomycetes | Tilletiales | Tilletiaceae | Tilletia | Puccinelliae |
| WA | Ascomycota | Sordariomycetes | Xylariales | Microdochiaceae | Microdochium |  |
| WA | Chytridiomycota | Spizellomycetes | Spizellomycetales | Spizellomycetaceae | Spizellomyces | Lactosolyticus |
| WA | Ascomycota | Sordariomycetes | Hypocreales | Nectriaceae | Fusarium | Oxysporum |
| WA | Basidiomycota | Ustilaginomycetes | Ustilaginales | Ustilaginaceae | Tranzscheliella |  |
| WA | Ascomycota | Dothideomycetes | Pleosporales | Didymosphaeriaceae |  |  |

Table S3. List of six most abundant arbuscular mycorrhizal species found in at least three samples in the field-collected soil samples of the MN and WA regions. For full species lists, refer to the data online (doi: 10.6084/m9.figshare.5926378).

| **Region** | **Order** | **Family** | **Genus** | **Specific epithet** | |
| --- | --- | --- | --- | --- | --- |
| MN | Glomerales | Glomeraceae | Glomus |  |  |
| MN | Glomerales | Claroideoglomeraceae | Claroideoglomus |  |  |
| MN | Glomerales |  |  |  |  |
| MN | Glomerales | Glomeraceae | Glomus | VTX00419 |  |
| MN | Diversisporales | Diversisporaceae | Diversispora |  |  |
| MN | Glomerales | Claroideoglomeraceae | Claroideoglomus | VTX00193 |  |
| WA | Glomerales | Glomeraceae | Glomus |  |  |
| WA | Glomerales | Claroideoglomeraceae | Claroideoglomus | VTX00193 |  |
| WA | Glomerales |  |  |  |  |
| WA | Paraglomerales | Paraglomeraceae | Paraglomus |  |  |
| WA | Glomerales | Claroideoglomeraceae | Claroideoglomus |  |  |
| WA | Paraglomerales | Paraglomeraceae | Paraglomus | VTX00335 |  |

Figure S1

Figure S1. Roots harboring structures typical of (a) arbuscular mycorhizal fungi or (b) non-mycorrhizal fungi referred to as dark septate endophytes that do not absorb the trypan blue stain and remain brown.

Figure S2

Figure S2. Sequence variant accumulation curves for (a) SSU and (b) ITS regions. Each line represents a sample.

Figure S3


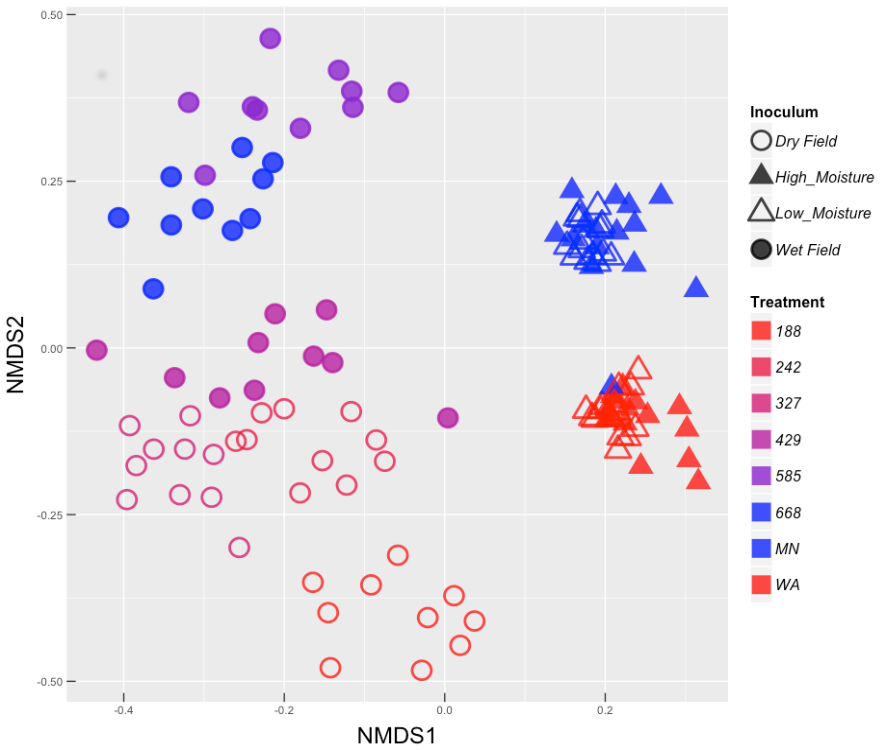


Figure S3. NMDS ordination of soil fungal communities based on sequence data from the field sites and greenhouse pots. Stress = 0.16. Field sites (circles) are coded by mean annual precipitation. Greenhouse pots (triangles) are coded by watering treatment.
